# HDR syndrome in a Japanese girl with biliary atresia: a case report

**DOI:** 10.1186/s12887-016-0550-9

**Published:** 2016-01-22

**Authors:** Yousuke Higuchi, Kosei Hasegawa, Miho Yamashita, Yousuke Fujii, Hiroyuki Tanaka, Hirokazu Tsukahara

**Affiliations:** 1 Department of Pediatrics, Okayama University Graduate School of Medicine, Dentistry and Pharmaceutical Sciences, 2-5-1 Shikata, Kita-ku, Okayama, 700-0914 Japan; 2 Department of Pediatrics, Okayama University Hospital, 2-5-1 Shikata, Kita-ku, Okayama, 700-0914 Japan; 3 Department of Pediatrics, Okayama Saiseikai General Hospital, 1-7-18 Ifuku, Kita-ku, Okayama, 700-8511 Japan

**Keywords:** *GATA3* gene, HDR syndrome, Hypocalcemia, Hypophosphatemia

## Abstract

**Background:**

Hypoparathyroidism, sensorineural deafness, and renal dysplasia (HDR) syndrome is an autosomal dominant disorder. We report the first detailed case of hypoparathyroidism complicated by biliary atresia.

**Case presentation:**

A 1-year-old Japanese girl was admitted to our hospital for living donor liver transplantation. She suffered from obstructive jaundice owing to biliary atresia. She also had persistent hypocalcemia. Despite oral calcium and abundant vitamin D supplementation, a laboratory test showed hypocalcemia (1.4 mmol/l) and hyperphosphatemia (2.6 mmol/l). The intact parathyroid hormone level was normal (66 ng/l) with severe vitamin D deficiency (25-hydroxy vitamin D: undetectable levels). There were no rachitic changes in metaphysis on X-rays. Her family history showed that her mother had sensorineural deafness, a low serum calcium level (2.1 mmol/l), hypoplastic left kidney, and a past history of an operation for right vesicoureteral reflux. We suspected that this patient and her mother have hypoparathyroidism, sensorineural deafness, and renal dysplasia syndrome. A heterozygous *GATA3* gene mutation (c.736delGinsAT) was found in this patient and her mother, but not in her father.

**Conclusion:**

This familial case confirms the importance of family history in the diagnosis of HDR syndrome. Regardless of marked vitamin D deficiency, the complication of hypoparathyroidism prevented the onset of vitamin D deficiency rickets in our patient.

## Background

The syndrome of hypoparathyroidism, sensorineural deafness, and renal dysplasia (HDR syndrome; OMIM: 146255) is an autosomal dominant disease [1]. Hypoparathyroidism causes symptomatic or asymptomatic hypocalcemia by normal or inappropriately low levels of parathyroid hormone (PTH) secretion [2]. Sensorineural deafness usually occurs bilaterally and the degree of symptoms varies from asymptomatic to severe hearing loss. Severity of renal involvement also varies from no renal abnormalities to renal dysplasia, hypoplasia, cystic kidneys, and vesicoureteral reflux.

HDR syndrome is caused by haploinsufficiency of the *GATA3* gene on chromosome 10p15. *GATA3* belongs to GATA transcription factor families, which are involved in vertebrate embryonic development of the parathyroid glands, inner ears, kidney, thymus, and central nervous system. *GATA3* contains six exons, and encodes two transactivating domains (TA1 and TA2) and two zinc finger domains (ZnF1 and ZnF2) [3].

Vitamin D is a fat-soluble vitamin that is absorbed from the intestines and requires bile acids for solubilization. Vitamin D malabsorption is common in jaundice patients with extra-hepatic biliary atresia (BA), even after hepatoportoenterostomy [4]. Vitamin D deficiency causes hypocalcemia, hypophosphatemia, and subsequently causes rickets in BA patients.

Here we report the case of a girl with HDR syndrome who presented with sustained severe vitamin D deficiency associated with BA, but no rachitic changes were observed.

## Case presentation

A 1-year-old Japanese girl was referred to our hospital for living donor liver transplantation from her father. She had the diagnosis of BA at the age of 2 months, when she presented with hypocalcemia (1.4 mmol/l; albumin-corrected calcium: 1.5 mmol/l; normal range: 2.0–2.8 mmol/l). She displayed no clinical signs of hypocalcemia, such as convulsion, muscle tremors, lethargy, and irritability.

Even though hepatoportoenterostomy had been performed 5 days after the diagnosis, progressive cholestasis required liver transplantation. Persistent hypocalcemia, which was considered to be associated with sustained vitamin D deficiency, occurred (25-hydroxy vitamin D [25OHD] was low [28.2 nmol/l, vitamin D deficiency is defined as <50 nmol/l]) [5]. After the operation, she was treated with oral calcium and vitamin D supplementation (alfacalcidol). Alfacalcidol is the active form of vitamin D3 and is used to treat vitamin D deficiency in Japan because the native form of vitamin D is not available for medical use. Despite the observation that serum calcium levels were low and serum phosphate levels remained at normal to high levels (1.8–3.1 mmol/l; normal range: 1.3–2.1 mmol/l), intact PTH levels were within the normal range (19–66 ng/l; normal range: 10–72 ng/l).

**Fig. 1:**
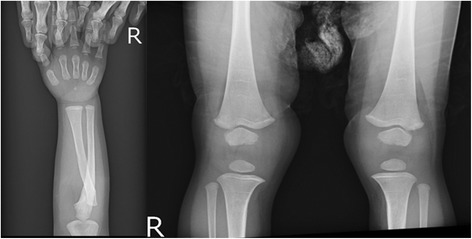
Radiographic image of the wrist and knee in the patient. No rachitic changes were observed in metaphysis

At the first visit to our department, under conditions of oral calcium and abundant alfacalcidol (3.0 μg/day), laboratory data showed marked hypocalcemia (1.4 mmol/l; albumin-corrected calcium: 1.5 mmol/l), hyperphosphatemia (2.6 mmol/l), and a normal intact PTH level (66 ng/l) with severe vitamin D deficiency (undetectable levels). The urine calcium to creatinine ratio was 0.15 mmol/mmol and tubular reabsorption of phosphate (%TRP) was 99 %. Additionally, there were no skeletal abnormalities on a physical examination and on X-rays (Fig. 1). These results suggested that she had hypoparathyroidism.

The patient’s height was 74.5 cm (-1.45 SD). Bone mineral density of the lumbar spine (L1–L4) measured by dual energy X-ray absorptiometry showed that the bone mineral density Z-score was −1.0.

Her family history showed that her mother had sensorineural deafness, hypoplastic left kidney, and a past history of an operation for right vesicoureteral reflux. Her mother’s serum calcium level was 2.1 mmol/l (albumin-corrected calcium: 2.1 mmol/l; normal range: 2.2–2.5 mmol/l) and intact PTH level was 55 ng/l (normal range: 10–65 ng/l). Further work-up by auditory brainstem response testing and abdominal ultrasonography showed that the patient had mild sensorineural left deafness and bilateral renal cysts. Based on these results, we strongly suspected that this patient and her mother have HDR syndrome and we conducted *GATA3* gene analysis.

**Fig. 2:**
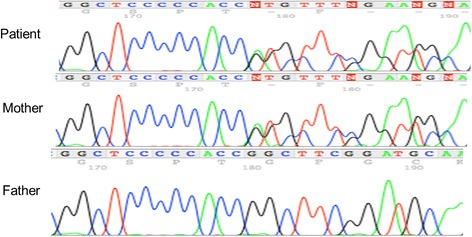
Sequence analysis of the *GATA3* gene. Electrochromatograms show that the patient and her mother had a heterozygous c.736delGinsAT mutation. The patient’s healthy father did not have the mutation

**Fig. 3:**
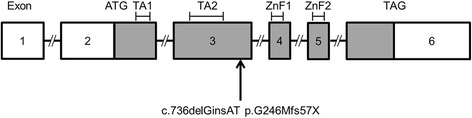
Schematic representation of the *GATA3* gene. The *GATA3* gene consists of six exons as indicated by boxes. Coding exons are shown in gray. The *GATA3* gene codes two transactivating domains (TA1 and TA2) and two zinc finger domains (ZnF1 and ZnF2). The coding region begins from exon 2 and the stop codon is located in exon 6. The arrow indicates the mutation site in our patient

Genomic DNA was extracted using a genomic DNA Extraction Kit (Qiagen Inc., Tokyo, Japan). PCR primers were designed to amplify all six exons and exon-intron boundaries of the *GATA3* gene. PCR products were purified with the QIAquick Purification Kit (Qiagen Inc.). Sequencing was performed with the BigDye terminator v3.1 Cycle Sequencing Kit and the ABI Prism 310 Genetic Analyzer (Applied Biosystems, Foster City, CA). The primer sequences for PCR are available upon request. Sequence analysis showed a novel heterozygous mutation in exon 3 of *GATA3*, c.736delGinsAT (Fig. 2), causing a frameshift with a premature stop codon after a new 57 amino acid sequence (p.G246Mfs57X) (Fig. 3). No other mutation was detected in the cording region of the *GATA3* gene. The same mutation was identified in her mother, but not in her father.

Before liver transplantation, 5 days of intravenous multivitamin supplementation (including cholecalciferol 200 IU/day) raised calcium levels from 1.8 to 2.3 mmol/l. After liver transplantation, cholestasis was resolved, but hypocalcemia and hyperphosphatemia were not improved. Therefore, oral calcium and alfacalcidol were required. At present, serum calcium and phosphorus levels are within the normal range by alfacalcidol (1.0 μg/day) alone.

## Discussion

We report the case of a girl with hypocalcemia due to BA. Further investigation showed HDR syndrome, which was confirmed by molecular analysis.

BA and HDR syndrome are rare diseases. To the best of our knowledge, only one patient who had BA and HDR syndrome has been reported, although the details were not described [6]. This is the first detailed report of hypoparathyroidism complicated by biliary atresia. Although there are only two cases, biliary atresia may be phenotypic variation of HDR syndrome. Moreover, there are a few reports about HDR syndrome with female genital malformation, but pelvic ultrasound test was not performed for the patient’s mother [7, 8].

The clinical characteristics of HDR syndrome are known to be heterogeneous. In HDR patients, 62.3 % exhibit the complete clinical triad, 28.6 % have hypoparathyroidism and renal disease, 2.6 % have deafness and renal disease, and 6.5 % have isolated deafness [9]. Although our patient’s sensorineural deafness is unilateral and is a mild abnormality, the triad was considered to be fulfilled. The exact prevalence of HDR syndrome is still unknown. There are likely to be some undiagnosed cases of this disorder among sensorineural deafness patients, such as the patient’s mother. In fact, symptoms present with varying degrees of severity, even in the same family with an identical *GATA3* mutation [9]. Because of this clinical heterogeneity, screening of *GATA3* mutations is useful for diagnosis and genetic counseling.

In this study, we identified a novel mutation of *GATA3* at exon 3 (c.736delGinsAT), causing a frameshift with a premature stop codon (p.G246Mfs57X). To the best of our knowledge, this mutation is novel and previously unpublished. The role of ZnF2 is essential for DNA binding, whereas the role of ZnF1 is stabilizing DNA binding affinity and interaction with other zinc finger proteins [3]. Ali et al. classified *GATA3* mutations into three classes as follows: class 1 is loss of DNA binding potential as a result of lack ZnF2 and represents more than 90 % of mutations reported in HDR syndrome; class 2 is a reduction in DNA binding affinity due to the mutation involving ZnF1; and class 3 is normal DNA binding and affinity, but possibly involves a conformational change [10]. In our case, the mutation resulted in truncated protein lacking ZnF2 and can be classified as class 1.

Rickets was reported to be found in 23 of 39 patients (59 %) with surgically unrepaired BA [11]. The pathogenesis of vitamin D deficiency rickets is as follows [12]. Vitamin D deficiency decreases intestinal calcium absorption. The resultant fall in circulating ionized calcium levels stimulates PTH secretion, which in turn causes an increase in renal phosphate excretion associated with an increase in mineral resorption from bone. With progressively increasing PTH concentrations, hypophosphatemia develops, which results in impairment of apoptosis of hypertrophic chondrocytes at the growth plate and mineralization of matrix vesicles in the osteoid. Histological and radiological features of rickets then develop. Collectively, excessive secretion of PTH and a hypophosphatemic state are crucial for establishment of vitamin D deficiency rickets [13].

Initially, researchers considered that hypocalcemia is secondary to vitamin D deficiency due to cholestasis and it can be treated by oral alfacalcidol and calcium supplementation. However, finding of hypocalcemia with hyperphosphatemia under a sustained severe vitamin D deficient status contradicts this mechanism.

## Conclusion

We identified a novel frameshift mutation of the *GATA3* gene in a patient with HDR syndrome and BA. Regardless of marked vitamin D deficiency, hypoparathyroidism prevented the onset of vitamin D deficiency rickets.

### Consent

Written informed consent was obtained from the patient’s parents for publication of this Case report and any accompanying images. A copy of the written consent is available for review by the Editor-in-Chief of this journal.

### Ethical statement

This study was approved by the institutional review board of Okayama University Hospital for clinical research. All procedures performed in studies involving human participants were in accordance with the 1964 Helsinki declaration and its later amendments.

## Abbreviations

BA: Biliary atresia
HDR: Hypoparathyroidism, sensorineural deafness, and renal dysplasia
PTH: Parathyroid hormone
TA: Transactivating domains
ZnF: Zinc finger domain
25OHD: 25-hydroxy vitamin D
